# Latent HIV Reservoirs in the Central Nervous System: Mechanisms, Barriers, and Therapeutic Approaches

**DOI:** 10.1021/acsinfecdis.5c01103

**Published:** 2026-03-26

**Authors:** Yohannes Matthew, Nicholas Foley, Daniel T. Claiborne, Zachary Klase, Alexej Dick

**Affiliations:** † Department of Biochemistry and Molecular Biology, 12312 Drexel University College of Medicine, Philadelphia, Pennsylvania 19102, United States; ‡ Department of Pharmacology and Physiology, Drexel University College of Medicine, Philadelphia, Pennsylvania 19102, United States; § Department of Biological Chemistry and Molecular Pharmacology, Blavatnik Institute, 1811 Harvard Medical School, Boston, Massachusetts 02115, United States; ∥ HIV Cure & Viral Diseases Center, The Wistar Institute, 3601 Spruce St, Philadelphia, Pennsylvania 19104, United States

**Keywords:** HIV-1 latency, central nervous system (CNS), blood-brain barrier (BBB), latency reversal agents (LRAs), RUNX1, TLRs, epigenetic regulation, immunotherapies, reservoir reactivation

## Abstract

Despite advancements in antiretroviral therapy (ART), HIV-1 remains incurable due to latent viral reservoirs. These reservoirs are located in distinct areas, such as the central nervous system (CNS). The CNS reservoirs flourish inside unique cell types, including myeloid cells such as microglia, perivascular macrophages, and astrocytes. These reservoirs are established early in infection, evade immune detection, and pose a significant challenge to the delivery of therapeutic agents. Although current ARTs can suppress viral transcription, the latently infected CNS cells can produce low-level persistent neuroinflammation and contribute to HIV-associated neurocognitive disorders (HAND). Multiple molecular mechanisms underlie the establishment and maintenance of CNS HIV reservoirs, including epigenetic modifications, transcriptional repression, and limited penetration of antiretroviral drugs across the blood–brain barrier (BBB). Specifically, latency involves transcriptional silencing through histone deacetylation and histone methylation, as well as the recruitment of repressive transcriptional complexes. Therapeutically targeting these mechanisms is critical for latency reversal and reservoir eradication. Two strategies, “shock and kill” and “block and lock”, take advantage of these mechanisms. The “shock and kill” method utilizes latency-reversing agents (LRAs) to stimulate transcriptional reactivation, exposing infected cells for immune clearance. Notably, several LRAs, including Vorinostat, JQ1, and Bryostatin-1, have been shown to penetrate the BBB and exhibit promising latency-reversal activity. However, their clinical efficacy is limited by incomplete reservoir reactivation and potential neurotoxicity. Emerging therapeutic targets, such as the transcription factor RUNX1, show significant promise for both potent HIV reactivation and lack of neurotoxicity. To enhance CNS reservoir targeting, novel strategies leveraging viral vectors or lipid nanoparticles are being explored. Overall, a comprehensive understanding of HIV-1 latency mechanisms in the CNS, coupled with the strategic development of BBB-penetrant, non-neurotoxic LRAs and adjunct immune therapies, is critical. Future therapeutic regimens will likely require a multifaceted approach to eradicate HIV-1 reservoirs safely and effectively within the CNS, ultimately progressing toward a functional cure.

## Graphical abstract

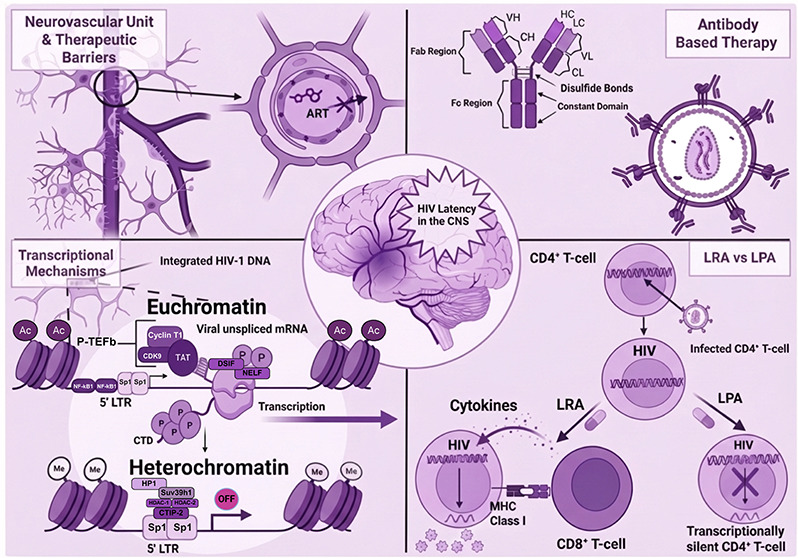

## Overview of HIV-1 Infection and Latent Reservoirs

HIV pathogenesis is highly complex, and more than 39 million people worldwide are currently living with HIV; consequently, a functional cure remains a major unmet need.^1,2^ Early antiretroviral therapy (ART) introduced in 1987 often failed to durably suppress viremia because of limitations, including rapid emergence of drug resistance, toxicity, and cost. In the early 2000s, the widespread adoption of combination ART enabled sustained viral suppression, transforming HIV into a manageable chronic condition and substantially improving the quality of life for millions of individuals.^2^ Despite the improved prognosis for people living with HIV (PLWH), there remain many burdens today, including the need for ongoing therapy and an increased risk of certain types of cancer, cardiovascular disease, and neurocognitive impairment.^3^ These burdens are likely driven by a complex interplay between the off-target toxicities of lifelong ART and the chronic inflammation fueled by low levels of viral transcription. The long-term effects of ART on the CNS have not been studied in HIV negative individuals for ethical reasons. As such, it is often hard to determine if observed adverse outcomes may be due to ART, HIV infection, or a combination of both. It is well established that adherence to ART and associated viral load suppression protect the CNS.^4,5^ However, evidence from other systems suggests that ART can cause neurotoxic effects.^6^ A well-characterized example is efavirenz, which has well-documented CNS toxicity, including neuropsychiatric symptoms such as dizziness, sleep disturbances, depression, and, in some cases, psychosis.^7^ While these CNS effects contributed to its declining use, efavirenz was ultimately replaced as a preferred first-line agent largely due to the superior virologic efficacy, higher barrier to resistance, and improved overall tolerability of integrase strand transfer inhibitors such as dolutegravir.^8^

Current ARTs are effective at suppressing HIV but cannot successfully eliminate HIV-infected cells from the host. Several classes of antiretroviral drugs have been developed and target different steps of the HIV life cycle, including fusion inhibitors, non-nucleoside reverse transcriptase inhibitors, nucleoside reverse transcriptase inhibitors, integrase inhibitors, protease inhibitors, and capsid protein inhibitors (Figure 1A). One recent example is Lencapavir, a novel, first-in-class, multistage, selective inhibitor of the HIV capsid protein. This long-acting subcutaneous injectable agent has demonstrated antiviral activity and has been used in individuals with extensive prior treatment exposure and in treatment-naïve PLWH. These therapies suppress HIV replication by targeting key steps in the viral lifecycle, thereby blocking infection of new target cells. Yet significant challenges remain, particularly within the CNS’s hidden reservoirs. Critically, ART does not eliminate the virus that has already integrated into the host cell genome, and latently infected cells that do not actively produce virus persist indefinitely as stable reservoirs.^9^

**Figure 1:**
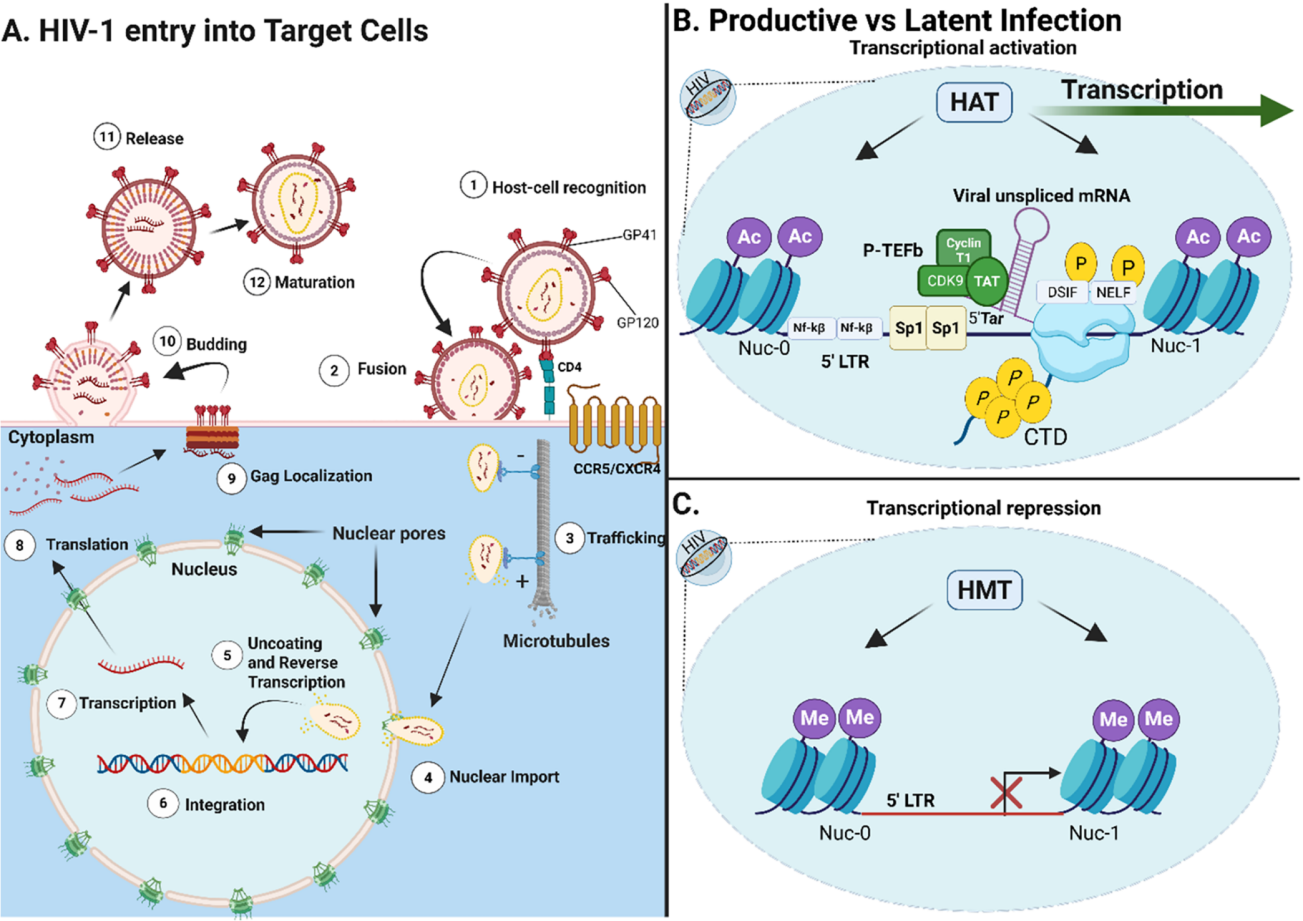
HIV-1 Entry and Transcriptional Regulation in Target Cells. (A) Overview of the HIV-1 replication cycle. Key steps in the HIV-1 replication include viral entry (fusion), integration into the host genome, transcription, translation into viral proteins, virion budding, and maturation. The illustration provides insights into the transcriptional activation mechanisms in actively infected CD4^+^ T-cells. (B) Histone acetyltransferases (HATs) incorporate acetyl groups to lysine residues on histones positioned at nucleosomes Nuc-0 and Nuc-1, promoting euchromatin formation and enhancing transcriptional accessibility. RNA polymerase II (RNA Pol II) assembles at the 5′ long terminal repeat (LTR) region of the HIV-1 genome, where associated factors contribute to the formation of the preinitiation complex (PIC). During transcription elongation, the HIV Tat protein binds to the trans-activation response (TAR) element of the nascent viral mRNA. This interaction recruits the positive transcription elongation factor b (P-TEFb) complex, which phosphorylates the DRB sensitivity-inducing factor (DSIF) and the negative elongation factor (NELF), thereby preventing transcriptional pausing. Additionally, the C-terminal domain (CTD) of RNA polymerase II undergoes phosphorylation, facilitating efficient transcription elongation. (C) Mechanisms of transcriptional repression in latent infection. In contrast to active transcription, histone methyltransferases (HMTs) add methyl groups to histone residues, thereby driving the formation of heterochromatin that limits transcriptional accessibility. Consequently, despite RNA polymerase II and the preinitiation complex (PIC) assembling at the HIV-1 LTR, transcription is effectively suppressed, maintaining viral latency. Therapeutic LRAs target these repressive mechanisms; for example, Vorinostat inhibits histone deacetylases (HDACs) to promote acetylation, JQ1 inhibits BRD4 to release P-TEFb, and other upcoming therapies inhibit transcriptional repression at the 5′ LTR. P: Phosphate, Ac: Acetyl group, Me: Methyl, HAT: Histone Acetyl Transferases, HMT: Histone Methyl Transferases. Created with BioRender.com.

While these therapeutics have been highly effective at suppressing viremia, they do not eliminate persistent HIV-1 reservoirs, particularly those in the CNS.^10^ The population of infected cells actively producing viral gene products decays after the start of ART, leaving a population of latently infected cells.^11^ These latently infected cells have HIV-1 proviral DNA integrated into their genome but remain transcriptionally silent.^9^ These long-lived reservoirs persist for the host’s lifetime and can lead to recrudescent viral replication and rebound if therapy is interrupted.^9^

This inability to eradicate latent reservoirs can lead to rebound viremia upon cessation of ART, as reactivation of virus from long-lived infected cells reseeds active systemic infection.^10^ HIV-1 latency is established early during infection and persists in resting memory CD4^+^ T-cells, partly due to transcriptional silencing by host epigenetic mechanisms (Figure 1B).^10^ In the CNS, this challenge is compounded by suboptimal antiretroviral drug penetration across the BBB, which allows latent infection to persist despite suppressed plasma viremia.^12^ Consequently, targeted strategies beyond standard ART, including novel latency-reversal and latency-promoting agents, are crucial for achieving sustained viral remission or cure. While newer long-acting agents such as lenacapavir, a first-in-class HIV-1 capsid inhibitor, have demonstrated high rates of durable virologic suppression and represent important advances in simplifying treatment delivery,^13^ they remain fundamentally limited by the same barrier as all current ART, the inability to eliminate stably integrated proviral DNA within latent reservoirs. This underscores the need for additional interventions specifically designed to target latent HIV.

Many HIV cure strategies aim to modulate the host transcriptional machinery to either durably silence proviral expression in infected cells (“block and lock”) or induce viral transcription to render infected cells visible to immune-mediated clearance (“shock and kill”). In addition, broadly neutralizing antibodies (bNAbs), discussed later in this review, represent a promising component of curative strategies because of the breadth of viral epitopes they can recognize. When paired with latency reversal, bNabs could enhance immune recognition and elimination of HIV-infected cells.

## Entry and Establishment of Latent Infection in the CNS

Building on knowledge of peripheral reservoirs, understanding how HIV-1 targets persist within CNS cells is critical. HIV-1 infects multiple cell types within the CNS, and the BBB creates unique challenges for viral eradication and effective drug delivery (Figure 2A).^14^ Two primary mechanisms have been proposed to explain how HIV-1 enters the CNS: (1) via the trafficking of infected lymphocytes through the meningeal lymphatic system, and (2) through the so-called “Trojan horse” mechanism, wherein monocytes that are precursors for brain macrophages are susceptible to becoming HIV-infected (HIV^+^) while crossing the BBB.^14^ Supporting the “Trojan horse theory”, Macrophages infected with HIV in the CNS can secrete pro-inflammatory cytokines and chemokines. These cytokines create a neuroinflammatory environment that recruits additional peripheral monocytes, thereby promoting viral entry into the CNS. This neuroinflammation also activates resident microglia, upregulating their CCR5 expression and thereby increasing their susceptibility to infection.^15^

**Figure 2:**
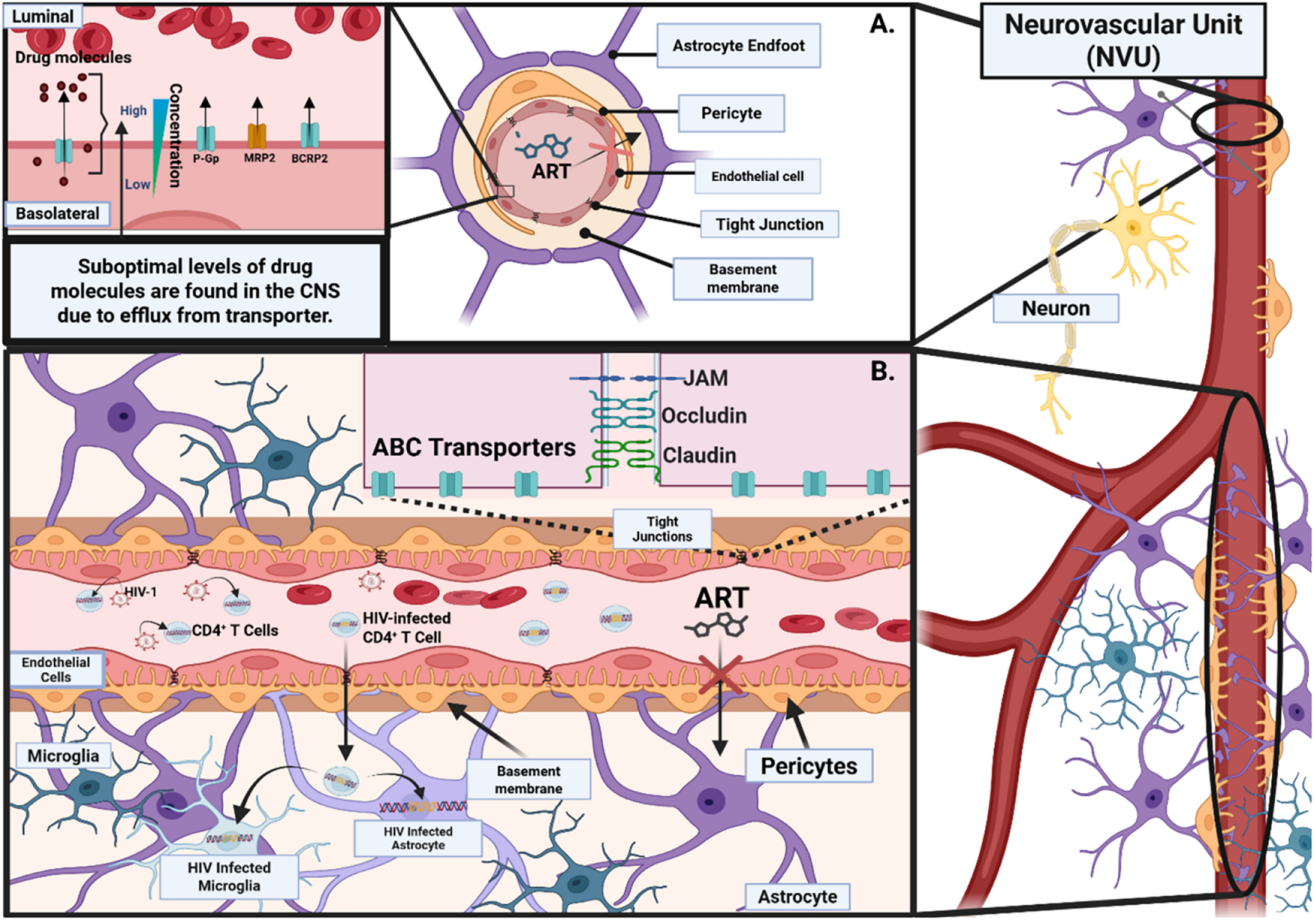
(A) Cross-sectional representation of the Neurovascular Unit (NVU), the Structural Features of the BBB, and HIV-1 CNS infiltration. This figure illustrates the neurovascular unit and highlights its key structural components, including brain microvascular endothelial cells (BMECs), pericytes, and astrocytic endfeet. The figure emphasizes the limited ability of antiretroviral therapy molecules to cross the BBB due to efflux by transporters such as P-glycoproteins (P-gp), multidrug resistance protein (MRP), and breast cancer resistance protein (BCRP) located on the luminal side of the BMECs. A clear concentration gradient is evident, with ART levels highest in the bloodstream and substantially lower in endothelial cells and the CNS. ART: antiretroviral therapeutic, P-Gp: p-glycoprotein, BCRP: breast cancer resistance protein, MRP2: multidrug resistance protein. (B) Overview of the organization and functional characteristics of the BBB, emphasizing its role in HIV-1 infection within the CNS. HIV-1-infected CD4^+^ T-cells cross the BBB via tight junctions between brain microvascular endothelial cells. Subsequently, CD4^+^ T lymphocytes infect astrocytes through a CD4-independent mechanism. Endothelial cells are closely associated with astrocytic end feet and pericytes, collectively maintaining the BBB’s selective permeability. The figure illustrates the limitations of antiretroviral therapy penetration into the CNS, highlighting how ART molecules are unable to cross the BBB, primarily due to the presence of tight junctions and active efflux transporters, ABC: ATP Binding Cassette. Created with BioRender.com.

Paradoxically, the exact BBB that fails to exclude HIV-1 also restricts the penetration of key antiretroviral drugs, making the CNS a difficult compartment to reach pharmacologically.^12^ For small-molecule compounds, passage across the BBB is influenced by molecular weight, lipophilicity, and plasma protein binding.^16^ Even when such compounds meet the necessary physicochemical criteria, ATP-binding cassette (ABC) efflux pumps (e.g., P-glycoprotein, organic anion transporters) in the CNS can further limit drug retention.^12,16^ As a result, the incomplete penetration of ART, alongside the establishment of the viral reservoir and latency, remains a significant barrier to HIV-1 eradication and may be another mechanism for inflammation linked to HAND.^17^

Once the peripheral virus has reached the CNS, HIV-1 can infect microglia, perivascular macrophages, and possibly astrocytes (Figure 2B).^12^ Entry into these cell types is mediated by the interaction of HIV gp120 with CD4 and a coreceptor, either CCR5 (R5-tropic virus) or CXCR4 (X4-tropic virus).^18^ The predominance of macrophages and microglia as CNS target cells indicates that R5-tropic strains are the principal variants that seed and persist in the brain. This has direct therapeutic implications, as CCR5 entry inhibitors such as maraviroc may have utility in strategies targeting CNS reservoirs. However, their efficacy depends on the tropism profile of the resident viral population.^18^

Critically, once established in the CNS, HIV undergoes independent viral evolution that is genetically distinct from the virus circulating in peripheral blood, consistent with the CNS functioning as a separate, compartmentalized reservoir.^19^ Phylogenetic analyses have demonstrated that CNS-derived viral sequences can diverge substantially from plasma-derived variants, likely driven by distinct selective pressures including differences in target cell availability, immune surveillance, and ART penetration.^19^ This compartmentalization has profound implications for cure strategies, as interventions that effectively target peripheral reservoirs may not be sufficient to eliminate virus persisting independently within the CNS.

The infection mechanism of myeloid cells in the CNS has been well established, but HIV infected astrocytes may also contribute to a persistent infection within the CNS (Figure 2B).^20^ These long-lived cells are typically thought to have lower levels of viral replication, perhaps reflecting the mechanism by which they are infected. In a recent study, Valdebenito et al. developed a comprehensive protocol to detect integrated HIV DNA, viral mRNA, and viral proteins within the frontal cortex and subcortical brain regions from PLWH on ART. By employing immunohistochemical staining techniques, the study identified a small but significant proportion of astrocytes infected with HIV-1, as demonstrated by the colocalization of markers such as Nef (an HIV DNA marker) with GFAP (an astrocyte-specific marker).^20^ Their findings indicate that GFAP-positive astrocytes constitute a notable component of the CNS viral reservoir, although levels of infected astrocytes were significantly lower in pre-ART samples than in long-term ART samples. Moreover, they observed that *in vitro* treatment with LRAs, such as suberoylanilide hydroxamic acid (SAHA) or Tumor Necrosis Factor α (TNF-α), transiently induced HIV-1 gene expression in infected astrocytes, resulting in a significant increase in viral mRNA and protein levels. Complementary *in vitro* experiments confirmed that astrocytes constitute a small but relevant population of HIV-infected cells capable of transmitting the virus. Notably, their study demonstrated that latently infected astrocytes can transmit HIV infection to CD4^+^ T-cells and macrophages via direct cell-to-cell contact.^20^ This cell–cell transmission depends on the formation of virological synapses, allowing for the immature budding of HIV particles from filopodial extensions.^21^ These insights underscore that direct intercellular contact is an efficient pathway for HIV dissemination among astrocytes, glial cells, myeloid cells, and lymphoid cells; thus, highlighting the complex dynamics underlying HIV persistence and spread in the CNS.^20^

After CNS infection, viral latency can be established through both preintegration and postintegration mechanisms, broadly analogous to those described in peripheral CD4^+^ T-cells.^22^ In preintegration latency, reverse-transcribed viral DNA remains unintegrated, often due to stalling of reverse transcription or nuclear import of the preintegration complex. In postintegration latency, proviral DNA is integrated into the host genome but remains transcriptionally silent, influenced by the chromatin environment at the integration site and the availability of host transcription factors that engage the HIV-1 LTR.^22^ However, CNS latency differs from the classical T-cell paradigm in several important aspects. In the periphery, the principal latent reservoir resides in long-lived resting memory CD4^+^ T-cells, where latency is primarily maintained through the quiescent transcriptional state of these cells.^22^ In the CNS, by contrast, the primary reservoir-competent cells are microglia and, potentially, perivascular macrophages, long-lived, self-renewing cells of myeloid lineage that are not replenished by peripheral monocyte turnover under homeostatic conditions.^23^ Microglia can harbor transcriptionally silent but replication-competent proviruses, representing a durable postintegration latent state.^23^

Importantly, the mechanisms sustaining latency in myeloid cells appear to differ from those in T-cells. Microglial latency involves distinct epigenetic landscapes, differential transcription factor availability (including restricted NF-κB and P-TEFb activity), and unique regulatory dynamics at the LTR that reflect the specialized biology of these resident CNS cells.^24^ Furthermore, the immune-privileged nature of the CNS, characterized by limited immune surveillance, the presence of the BBB, and reduced cytokine-mediated activation signals, creates a microenvironment that may independently favor the maintenance of viral latency in ways not recapitulated in the periphery.^23^ These distinctions underscore that strategies developed to reverse latency in peripheral T-cell reservoirs cannot be assumed to be equally effective in the CNS.

## Genetic Diversity of HIV in the CNS

In patients with undetectable plasma viremia, integrated HIV persists in distinct tissue reservoirs, including in the CNS.^25^ Discontinuing ART in the presence of residual reservoirs can trigger rapid viral rebound, making medication adherence an indefinite commitment.^26^ A key obstacle to reversing HIV latency in the CNS is its tremendous genetic variability.^14^ HIV reverse transcriptase lacks 3′-5′ proofreading, resulting in an error rate of 1 in 1700–2000 nucleotides, which is significantly higher than that of DNA polymerases (10^–6^ to 10^–4^).^27^ This high mutation rate enables the virus to adapt to diverse compartments, including blood, lymph nodes, the genital tract, organs, and the CNS/CSF.^28^ SIV studies in rhesus macaques have shown that infection can reach the CNS in as few as 10 days, allowing great viral diversity to be reached later in infections.^28^

At the mechanistic level, LTR sequence variations can affect the efficacy of transcription factors such as SP1-III and NF-kB.^29^ Additionally, polymorphisms, including ETS1, AP1, and C/EBP, tend to accumulate in CNS HIV.^9^ These findings underscore the need to reverse latency through multiple pathways, which current clinical trials for LRAs often fail to address. These could explain the decreased efficacy of current LRAs *in vivo* compared to their *in vitro* performance.

## The CNS as a Critical Barrier to HIV Eradication

Despite the effectiveness of ART, HIV infection remains a major public health challenge because persistent viral reservoirs in distinct tissue compartments are not eliminated by ART.^30^ While the most widely studied reservoirs are CD4^+^ memory T-cells, CNS reservoirs are thought to primarily comprise infected myeloid cells, such as perivascular macrophages and microglia,^3,9,30^ and astrocytes.^20,21^ The persistent infection of potentially multiple cell types in the CNS creates protected reservoirs in this compartment that persist even under ART administration.^31^ The continued existence of CNS and other reservoirs provides a pool of replication-competent viruses, leading to viral rebound after cessation of therapy and possibly the development of escape mutations should replication suppression be incomplete.^32^ Persistently infected cells in the CNS lead to a range of neuropathologic, behavioral, and cognitive effects collectively known as HIV-Associated Neurocognitive Disorders (HAND).^33,34^ In the ART era, HAND remains prevalent even in virally suppressed individuals and is thought to be driven by chronic neuroinflammation,^35^ which is itself driven primarily by the low-level, persistent infection in CNS populations.^36^ HIV enters the CNS within the first weeks of infection, and resident microglia, macrophages, and astrocytes become infected before individuals are placed on suppressive ART.^37^ The latent CNS cells are generally long-lived, resistant to HIV-induced apoptosis, and do not divide,^38^ suggesting that the CNS reservoir may be as stable and long-lived as that found in memory CD4^+^ T-cells in the periphery.

Furthermore, due to their long lifespan, each infected macrophage, microglia, or astrocyte may continue to produce the virus long after the initial infection.^38^ This highlights some critical distinctions, for example, CD4^+^ T-lymphocytes undergo clonal expansion or viral lysis, resulting in shorter-term viral reservoirs. In contrast, myeloid lineage cells are resistant to HIV-mediated lysis, enabling them to serve as long-term, productive reservoirs.^39^

## Mechanisms of HIV Transcriptional Control

Next, we consider the CNS and the fundamental mechanisms that drive HIV transcription, with an emphasis on Tat-dependent and Tat-independent pathways and how these processes may be leveraged to disrupt latency. HIV transcription is regulated by multiple mechanisms, which vary depending on the viral phenotype and cell type. Most notably, the viral transactivator of transcription protein (Tat) exerts control by binding the HIV TAR element, an RNA stem-loop at the 5′ end of all viral transcripts.^40^ Once Tat is bound to TAR, it recruits P-TEFb, which is comprised of CDK9 and cyclin T1, thereby enhancing the processivity of RNA Polymerase II (RNA Pol II) and leading to more LTR-mediated transcription.^41^ Epigenetic modification of the integrated promoter, including deacetylation and methylation, hinders access to the promoter,^42^ which in turn stalls RNA polymerase II (RNA Pol II) and generates short, incomplete transcripts.^43^ In this state, little to no Tat protein is produced. Early in infection and in some latent states, Tat-independent transcription initiates low-level replication, especially during latent stages.^41^

The HIV promoter within the LTR is critical for latency and replication, as it contains binding sites that initiate transcription by recruiting transcription factors and assembling the RNA polymerase II complex. Initiation of HIV transcription is regulated by post-translational modification of histone tails, alterations in nucleosome placement along the LTR, recruitment of cellular proteins that alter the chromatin environment, the availability of Pol II, and the recruitment of Tat.^44^ Latency is maintained, in part, by epigenetic activities such as DNA methylation, hypermethylation, and histone hypoacetylation that package the viral DNA, as well as by proteins that reinforce this state.^45^ Processive transcription, wherein Pol II becomes capable of high-speed production of full-length transcripts, is mediated by the recruitment of P-TEFb (CDK9/Cyclin T1) by the Tat protein. In the absence of P-TEFb, HIV transcription is abortive, leading to the production of only short transcripts that span the U5 region of the LTR. The methylation of histone tails and disruptions in the access of epigenetic remodelers and transcription factors to LTRs can decrease or block viral transcription. Thus, perturbations in either the cellular or Tat-dependent activation phases can suspend viral transcription.^40,46^

HIV transcription is closely linked to the activity of host CD4^+^ T-cells,^47^ while T-cell receptor (TCR) stimulation typically boosts viral transcription.^48^ Agents that promote broad immune stimulation can significantly increase the transcription of HIV from latent cells and can produce detrimental side effects, including inflammation and autoimmunity.^48^ Certain LRAs (e.g., JQ1) exhibit anti-inflammatory effects, dampening T-cell activation, while paradoxically promoting HIV transcription.^49^ These observations underscore the genetic and phenotypic diversity of HIV’s latency mechanisms, suggesting that multiple pathways must be targeted to fully reactivate and clear a significant portion of the latent reservoir.^50^ Such intricate control over viral gene expression also illustrates the breadth of factors that can be leveraged for latency reversal, including host transcription regulators.

Latency also relies on epigenetic activities, such as DNA methylation and histone acetylation, which package the viral DNA, as well as the presence of other host proteins that reinforce this state.^45^ These chromatin modifications occur on the lysine residues of the histone 3 (H3) tail. As a result, many latency reversal agents target these chromatin modifications, creating a relatively decondensed state of DNA (euchromatin) that ultimately allows the transcription machinery to bind to the HIV promoter.

The integration site of HIV also influences the dynamic control of HIV within the host genome. Integration into euchromatin, a loosely packed DNA conformation, is associated with higher transcriptional activity, whereas heterochromatin, a tightly packed structure, restricts transcription.^51^ HIV preferentially integrates into DNA near nuclear pores, which contain actively transcribed genes.^52,53^ However, integration into genes deeper within the nucleus is possible and may help establish the latent reservoir. These deep nontranscribed genes, often referred to as gene deserts (nonprotein-coding regions), represent a minority of infections and exhibit remarkably low levels of HIV transcription. As such, HIV latency is shaped by the host cell’s epigenetic landscape, and many current LRAs leverage this by manipulating host deacetylation or methylation pathways.^54^

The studies discussed above primarily focus on HIV transcription in CD4^+^ T-cells; in contrast, the mechanisms regulating transcription in myeloid populations are less well-defined, further complicating our understanding of HIV latency. Many similarities have been identified in the transcriptional profiles of myeloid and CD4^+^ T-cells. Exogenous stimuli that activate nuclear factor kappa B (NF-κB), including cocaine exposure or stimulation of toll-like receptors, activate transcription in myeloid cells, the same as in CD4^+^ T-cells.^40^ Acetylation or methylation of lysines in the histone H3 tail, DNA methylation, activity of histone deacetylases (HDAC1/2/3), and methyltransferases also play a role in the latency of myeloid cells.^55^ Additionally, bromodomain-containing protein 4 (BRD4) can also suppress myeloid HIV transcription.^56^ Runt-related transcription factor 1 (RUNX1) may also regulate HIV transcription in myeloid cells, including in the context of benzodiazepine-induced transcriptional effects. However, myeloid cells can exhibit distinct regulatory mechanisms, such as the HDAC1/2-containing CoREST-complex. Notably, these factors were not identified in a functional screen of transcriptional networks controlling HIV transcription in T-cells, suggesting myeloid-specific regulation and mechanistic divergence in how HIV transcription is controlled across cellular reservoirs.^57^

## Therapeutic Challenges in the CNS

Central to the challenge of reversing latency in the CNS is the BBB, a specialized structure that regulates molecular and cellular trafficking between peripheral circulation and the CNS.^58^ The BBB represents a specific barrier to the delivery of therapeutics to the brain. The CNS, particularly neurons and glia, has exceptionally high energy demands, requiring tight regulation of cerebral blood flow, orchestrated by the neurovascular unit (NVU) (Figure 2A).^58^ This NVU comprises brain parenchymal cells, endothelial cells, astrocytes, and pericytes, which work in concert to maintain a biochemical gradient between the CNS and peripheral tissues. Within the NVU, brain microvascular endothelial cells (BMECs) form the BBB’s innermost layer, sealed by tight junctions and largely devoid of fenestrations (Figure 2A). Astrocyte end feet envelop these BMECs on the parenchymal side, modulating junctional integrity and acting as an additional barrier to molecular transport.^16^ Pericytes also regulate the proliferation and survival of BMECs and facilitate crosstalk between endothelial cells and astrocytes. Together, these components restrict the entry of pathogens and many small-molecule therapeutics, thereby limiting CNS exposure to ART.^12^

ART itself provides a clear example of how the BBB influences therapeutic choices. ART concentrations in the brain are often substantially lower than those achieved in peripheral blood (Figure 2A).^12^ In addition, several ART regimens can produce neuropsychiatric adverse effects, including depression, cognitive deficits, and sleep disturbances.^59^ Although no longer recommended as a first-line agent in many settings, Efavirenz is a well-described example and has been associated with vivid dreams, insomnia, anxiety, and psychosis.^59^ Emerging evidence also suggests that ART exposure is not uniform across the CNS. A study by Wang et al. identifies new mechanisms that may explain why successful ART therapy may not be sufficient to prevent the onset of HAND. Common ART drugs, including dolutegravir, tenofovir, lamivudine, and efavirenz, were found to have differing concentrations across different brain regions in Ugandan decedents, with a median time from death to autopsy of 8 h.^60^ These findings imply that CSF drug levels may not reliably reflect drug exposure within specific brain parenchymal compartments, and that regional pharmacokinetic heterogeneity could meaningfully shape viral persistence. Such spatial gradients in drug concentrations may contribute to ongoing HIV compartmentalization and genetic diversification within the CNS. Regions with low effective drug exposure could permit residual replication or transcriptional activity, creating conditions that favor selection of drug-resistant variants, particularly in the setting of suboptimal adherence.This is consistent with the persistence of HAND in the post-ART era. For example, abacavir concentrations have been reported r to range from 5.2 to 10.9 μM in plasma but only 0.5 to 1.8 μM in cerebrospinal fluid.^12^ Such steep exposure diverences underscore the ned for lRAs and other therapeutic modalities that achive effective CNS penetration while minimizing neurological toxicity.

## Heterogeneous Reservoirs and the Need for LRAs

To fully appreciate the need for new agents, it is crucial to recognize the scope of HIV’s compartmentalization and the potential role of LRAs in targeting transcriptionally silent proviruses. During early infection, HIV integrates into host CD4^+^ T-cells, replicates, and migrates to multiple compartments, including the CNS, cerebrospinal fluid (CSF), gut-associated lymphoid tissue (GALT), lymph nodes, spleen, bone marrow, blood, and the genital tract.^61^ This widespread migration results in heterogeneous viral reservoirs that can diverge genetically across anatomical sites.^30^ In contrast to ART, which successfully stalls viral transcription and reduces plasma virus to undetectable levels, LRAs specifically target the transcriptionally silent provirus for reactivation.^30^ LRAs have therefore been proposed as a curative component of “shock-and-kill” strategies, aimed at promoting viral antigen expression and enabling the clearance of reservoir cells.

These distinct compartments host different cellular compositions and immune microenvironments, which likely shape both viral tropism and reservoir maintenance. In the brain, approximately 5–10% of cells are microglia and other myeloid-lineage populations, suggesting that macrophage-tropic (M-tropic) or myeloid-adapted viruses could be prominent. Consistent with this, HIV DNA has been detected in CD68/CD11b-positive microglia/macrophages in post-mortem brain tissues.^62^ However, humanized mouse models lacking human myeloid cells also demonstrate that T-cells alone establish and maintain HIV levels in the brain.^63^ Notably, the interpretation of these studies can depend on the timing of the experiment (for example, the number of weeks postinfection at which brains are harvested). Taken together, a parsimonious model is that infected T-cells may seed CNS infection early. At the same time, long-term maintenance of the CNS reservoir may shift toward myeloid or glial populations in a patient-dependent manner, influenced by viral tropism and local selective pressures.

Despite the promise of LRAs, the biological understanding of HIV transcription remains complex and varies with viral phenotype and infected cell type. There is evidence that HIV LTRs taken from the CNS often have a multitude of polymorphisms in transcription factor motifs.^64^ This frequently leads to decreased transcriptional activity and reduced responsiveness to some LRAs.^64^ These findings underscore the need to reverse latency through multiple pathways, which current clinical trials for LRAs often fail to address. This could also explain the decreased efficacy of current LRAs *in vivo* compared to their *in vitro* performance.

## The Unmet Need for BBB-Penetrant LRAs

LRAs are a central component of the proposed “shock and kill” strategy; however, comparatively little emphasis has been placed on achieving drug delivery to difficult-to-access compartments, such as the CNS. Accordingly, a key step toward eliminating latently infected cells is the development of LRAs with demonstrable CNS penetration that can safely induce viral reactivation while minimizing neurotoxic risks.

The initial seeding of HIV across different brain regions may contribute to the severity of HAND. Notably, PLWH who initiated ART during chronic infection exhibit higher rates of cognitive impairment than those who started ART during primary infection, closer to the time of exposure, even after achieving durable viral suppression.^65^ Table 1 summarizes representative ART agents and their relative potential for BBB penetration, underscoring a central challenge: to eliminate CNS reservoirs via “shock and kill”, LRAs must reach relevant brain compartments; conversely, for “block and lock”, transcriptional silencing agents must achieve sufficiently broad CNS distribution to suppress proviral activity across regions. Developing CNS-permitting LRAs with acceptable safety profiles is therefore critical for improving control over, and ultimately clearing, reactivated virus. Although some LRAs (e.g., Ro5–3335, bryostatin-1, and Vorinostat) show evidence of BBB permeability, most were not explicitly designed for CNS delivery, and systematic evaluation of CNS pharmacokinetics remains limited.^66^

**Table 1:** Antiretroviral Therapies and Latency Reversing Agents:

**ART**	**plasma concentration**	**cerebrospinal fluid (CSF) concentration**	**refs**
**maraviroc** (CCR5 inhibitor)	21.4–478.0 ng/mL	1.83–12.2 ng/mL	^67^
**enfurvirtide** (entry inhibitor)	3.7 μmol/mL	not determined	^12,67,68^
**nevirapine** (non-nucleoside reverse transcriptase inhibitor - NNRTI)	7.5–16.9 μmol/mL	1.3–10.9 μmol/mL	^12,67,68^
**raltegravir** (integrase inhibitor)	37.0–5180.0 ng/mL	2.0–126 ng/mL	^67^
**abacavir** (nucleoside reverse transcriptase inhibitor - NRTI)	5.2–11.0 μmol/mL	0.5–1.8 μmol/mL	^12,67,68^
**indinavir** (protease inhibitor)	12.2–13.0 μmol/mL	0.03–0.66 μmol/mL	^67^

**LRA**	**plasma concentration**	**cerebrospinal fluid concentration**	**refs**
**disulfiram** (disulfide) depletes PTEN levels. this prevents increased AKT phosphorylation and activation of a signaling pathway that leads to latent HIV-1 expression.	rapidly reduced in blood to diethyldithiocarbamate (DDC) within minutes (*in vitro*)	preclinical distribution: brain shows the lowest/least detectable levels in early distribution studies.	^69–71^
**alprazolam** (benzodiazepine) inhibitor of the RUNX1 transcription factor that negatively regulates HIV-1 transcription. Potentiates STAT5 recruitment to the viral promoter.	blood (median) 0.024 mg/kg	brain (median): 0.059 mg/kg (brain:blood ratio (median): 2.21)	^72–75^
**decitabine** (methyltransferase inhibitor) Blocks the addition of methyl groups, which modulates the expression of HIV after the addition of lysine residues on histone.	∼1.3–1.6 μM (standard i.v. dose in humans)	lower than plasma, typically 27–58% of plasma levels in animal models.	^76–79^
**vorinostat** (SAHA) (HDAC inhibitor) Can inhibit Histone Deacetylase Activating Complex (HDAC), which allows for the binding of RNAPII (RNA Polymerase II) and subsequent transcriptional activation.	plasma *C*_max_ (oral 400 mg) ∼1.2 μM	in the ventricular-CSF sampling cohort: mean CSF ∼ 75.4 nM	^69,80–83^
**JQ1** (BRD4 inhibitor) BRD4 agonist acts as an inhibitor of the BET family of proteins. Specifically, JQ1 prevents BRD4 from binding to the HIV promoter, thereby allowing Tat to recruit and stimulate HIV expression.	plasma *C*_max_ 34 μg/mL at 15 min after 50 mg/kg i.p. in mouse PK.	AUC_brain_/AUC_plasma_ = 0.98 (98%) after 50 mg/kg i.p.	^84–86^
**Bryostatin-1** (PKC (protein kinase C) agonist) activates protein kinase C (PKC) alpha and delta. Stimulated the transcription of the LTR by activating the transcription factor NF-kB.	plasma *C*_max_ (mouse 15 μg/m^2^ i.v. tail vein) ∼ 2.5 nM	peak brain concentration ∼ 0.2 nM at ∼1 h postdose; peak brain concentrations >8% of peak blood plasma.	^87–89^

## Examples of HIV BBB-Permeable LRAs

### Vorinostat (Suberoylanilide Hydroxamic Acid) (SAHA)

A class I HDAC inhibitor initially developed for T-cell lymphoma.^80^ By inhibiting Class I and II HDACs, Vorinostat relaxes chromatin in the HIV LTR, allowing promoter elements to bind more effectively.^90^ Its proven BBB penetration makes Vorinostat one of the more promising CNS-directed LRAs. However, its effects on other cell types (e.g., microglia and macrophages) remain largely uncharacterized.^23^ Vorinostat has been shown to have significant uptake into brain tissue, with concentrations reaching 5–7 times those in blood in mice.^91^ This effect is thought to arise from altered P-glycoprotein activity, which regulates drug efflux at key CNS barriers.^91^

### Bryostatin-1

A natural PKC agonist from the marine bryozoan Bugula neritina. PKC activation phosphorylates and inactivates IκB, thereby liberating NF-kB to upregulate HIV transcription.^88^ Combining PKC activation with histone acetylation or RUNX1 inhibition may produce synergistic effects.^92^ It was shown that Byrostatin-1 could cross the BBB in clinical trials as an Alzheimer’s medication.^93^

### JQ1 (a BET Inhibitor)

Known to cross the BBB with up to 98% efficacy, JQ1 targets the bromodomains of proteins that regulate transcription of HIV.^49^ In addition to downregulating T-cell activation genes, JQ1 enhances HIV transcription by competitively binding to BRD4, thereby freeing P-TEFβ from a BRD4-P-TEFβ complex. This resumes Tat-mediated transcription of HIV.^49^ JQ1 may have roles in SIRT1/RelA downregulation, HEXIM1 upregulation, and Myc suppression; however, these roles require further investigation.^49^

### Disulfiram

A unique NF-kB activator commonly used in the treatment of chronic alcoholism. In addition to inhibiting aldehyde dehydrogenase, it can also deplete PTEN, thereby increasing AKT signaling. This results in an increased NF-kB level, which can bind to the HIV LTR and stimulate transcription.^94^

## Current HIV-1 Cure Strategies and Their Limitations in the CNS

### “Shock and Kill” Approaches

Among HIV cure attempts, “shock and kill” and “block and lock” strategies offer distinct routes to confronting entrenched HIV latency. One prominent strategy to eliminate latent HIV-1 reservoirs is the “shock and kill” approach, in which LRAs reactivate quiescent proviruses, producing viral proteins and facilitating immune-mediated clearance of infected cells (Figure 3).^66^ Multiple drug classes are classified as LRA’s, and epigenetic modifiers include histone methyltransferase and histone deacetylase inhibitors, which respectively prevent the addition of repressive marks and prevent the removal of active marks from proviral DNA.^66^ Additional LRAs target intracellular signaling pathways, such as protein kinase C agonists.^66^ They also function as cytokine/immune receptor agonists, stimulating infected cells to produce viral antigens.^66^

**Figure 3:**
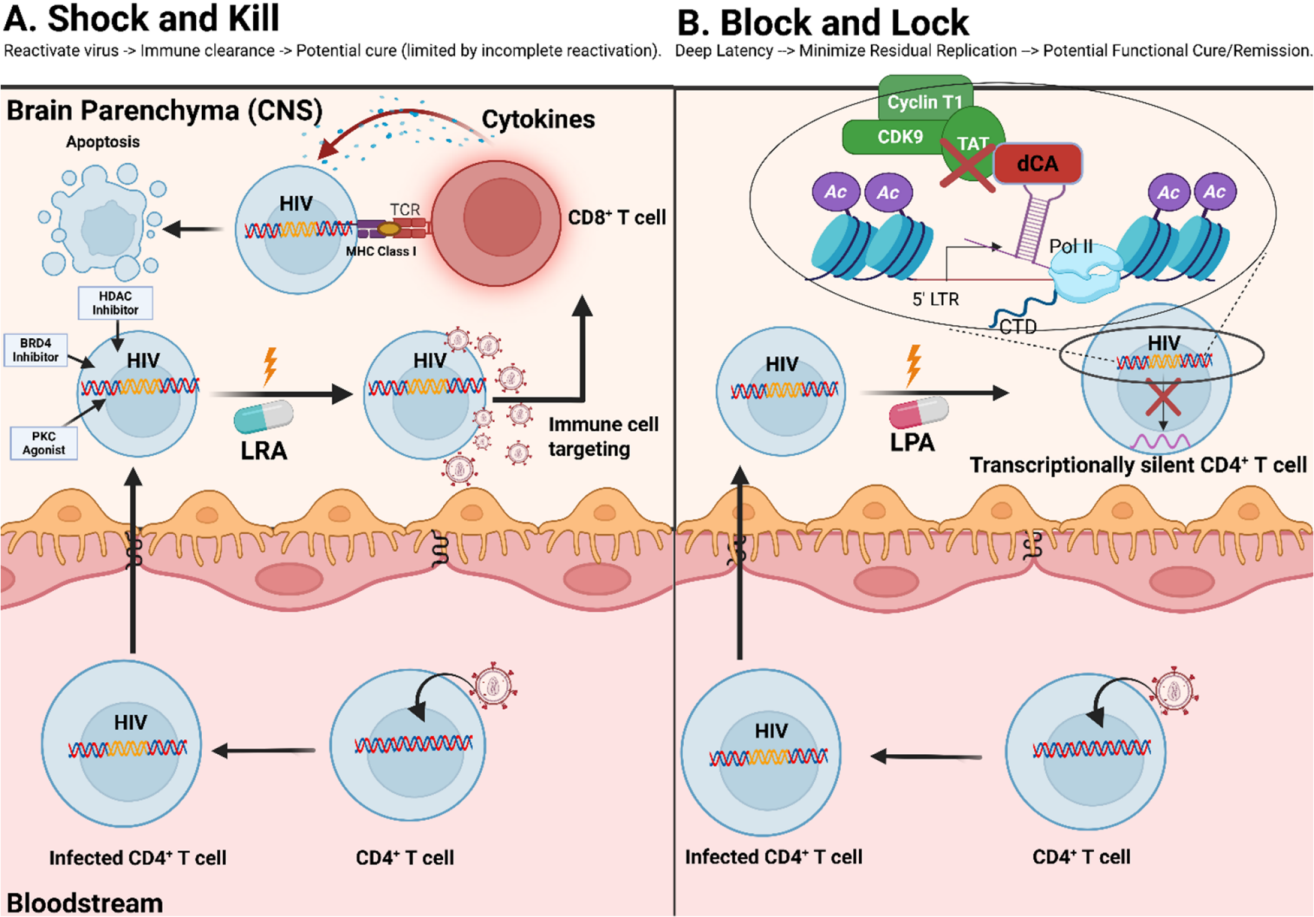
Contrasting Paradigms of HIV Latency Management: LRAs (″Shock and Kill″) versus LPAs (″Block and Lock″). Illustration of two distinct therapeutic strategies for managing HIV latency. (A) (″Shock and Kill″) depicts a latently infected CD4^+^ T-cell undergoing viral reactivation upon receiving an LRA signal. Following reactivation, an immune effector cell (specifically, a CD8^+^ T-cell) recognizes and targets the infected cell, inducing apoptosis and clearance. (B) (″Block and Lock″) illustrates an alternative strategy, where a latently infected CD4^+^ T-cell receives a latency-promoting agent (LPA) signal, reinforcing transcriptional silencing. This mechanism highlights the inhibitory action of the compound didehydro-cortistatin A (dCA) on critical HIV transcription elongation factors, including Tat, CDK9, and Cyclin T1, thereby maintaining durable latency without inducing viral production. LRA: latency reversal agent, LPA: latency promoting agent, BRD4: Bromodomain 4, MHC: Major histocompatibility class I, TCR: T cell receptor, PKC: Protein Kinase C, HDAC: Histone Deacetylase Inhibitor, dCA: didehydro-cortistatin A, CDK9: Cyclin-dependent kinase 9, Ac: Acetyl group. Created with BioRender.com.

Due to the mechanistic diversity of HIV latency, LRAs have shown limited efficacy *in vivo*. Incomplete reactivation of latent viruses often reflects the heterogeneity of reservoir cell subsets. However, since many latency reversal agents are T-cell specific and T-cell infection is rare in the CNS, data on how LRAs behave in the CNS reservoir cell subsets are limited, and future research should investigate whether LRAs are indeed failing due to the diverse mechanisms of latency in these CNS cell types.

The translation of many “shock and kill” approaches from preclinical studies to clinical trials remains constrained by several factors. Addressing these limitations is essential to establishing a viable therapeutic pathway toward an HIV cure.

- Adequate clearance of HIV in the CNS must be established. Reactivation of viral transcription in the CNS without proper viral clearance may come with detrimental side effects on neuronal health and could lead to HIV-associated neurocognitive disorders.^14^ Identification of LRAs that can cross the BBB and effectively clear the virus from the CNS is critical to advancing “shock and kill”.
- Combinations and interactions between different LRA classes should be investigated. Targeting multiple pathways involved in latency may be necessary to fully reactivate the virus in diverse cell types. Establishing more diverse *in vitro* models could benefit this. Further research on combinations should investigate the efficacy of latency reversal across multiple cell models, potential drug interactions, toxicity, and the impact of these therapies on the host’s immune system.
- Identification of robust *in vivo* biomarkers of latency reversal may be critical in evaluating efficacy. At present, HIV RNA measurements are commonly used to assess LRA activity. However, RNA induction may not reliably reflect full productive transcription or virion release. His limitation was highlighted in clinical studies of vorinostat, where increases in HIV RNA did not translate into measurable changes in free virus, potentially limiting immune recognition and clearance.

If these limitations can be addressed, a clinically effective shock-and-kill strategy could be rigorously tested in trials.

### “Block and lock” Approaches

It is essential to consider the long-term silencing of HIV-1 as an alternative approach to achieving ART-free viral suppression. In contrast to many of the “shock and kill” approaches widely applied here, “block and lock” strategies offer a reasonable alternative, as various latency-promoting agents can drive the virus into a deep state of latency by targeting a few host-specific mechanisms (Figure 3).^95^ Such latency-promoting agents (LPAs) typically target host epigenetic regulators or interfere with key viral proteins required for transcription (Figure 3).^95^ For example, LEDGINs (lens epithelium-derived growth factor inhibitors) bind HIV-1 integrase and alter its chromatin integration patterns, shifting the provirus away from actively transcribed regions. Topotecan, a camptothecin analog, has also been reported to modify the epigenetic landscape by promoting intron retention and upregulating SPF6 expression, thereby altering splicing by increasing the number of unsliced transcripts. This was initially reported in a study that applied topotecan to infected primary CD4^+^ T-cells and found that it impaired HIV-1 transcription.^96^ Another well-studied LPA is didehydro-cortistatin A (dCA), which inhibits Tat, the viral transactivator critical for robust HIV-1 transcription.^97^ By blocking the Tat-mediated recruitment of P-TEFb, dCA reduces HIV-1 RNA production and p24 antigen expression.^97^ In *ex vivo* studies with CD4^+^ T-cells from virally suppressed individuals, dCA consistently prevented viral reactivation under stimulatory conditions.^97^

Such deep latency-inducing approaches may be particularly appealing for CNS infections, where lower drug penetration and immune surveillance make robust shock-and-kill clearance difficult. Sustained silencing could, therefore, represent a viable path to long-term HIV control in the CNS. Moreover, increasing Tat production is involved in HIV’s neurotoxicity.^98^ In one study, Li et al. investigated the potential of dCA to alleviate HIV-related neuropathogenesis and found that dCA inhibited the release of inflammatory signaling proteins, such as interleukin-1β and TNF-α, within an astrocytic cell line.^98^ Ultimately, such approaches could improve Tat-associated outcomes in HAND and the overall quality of life for those patients, regardless of achieving a complete cure.^98^ Understanding the mechanisms of transcriptional control across different cell types may be critical for developing next-generation block-and-lock strategies. Peteres and Stevenson reported that NF-kB inhibition promoted loss of proviral competence in macrophages.^99^ In their work, they emphasized that myeloid cells maintain viral infections in people with HIV-1 and showed that macrophages from ART-suppressed individuals released replication-competent virus following exposure to PAMPs such as lipopolysaccharide (LPS).^82,99^ Using a refined macrophage model of HIV-1 latency, they treated infected macrophages with clinically relevant concentrations of NF-kB inhibitors caffeic acid and resveratrol, and then assessed downstream inflammatory signaling, including interleukin-10 production. Inhibition of interleukin-10 by caffeic acid and resveratrol was consistent with NF-kB pathway suppression and supported the conclusion that NF-kB inhibition can render latent HIV-1 genomes in macrophages refractory to reactivation.^99^ Nevertheless, both “shock and kill” and “block and lock” approaches remain limited by suboptimal penetration into the CNS and incomplete efficacy within the CNS microenvironment, motivating the pursuit of more specific and more deliverable molecular targets.

## Novel Targets for Latency Reversal in the CNS

To date, reactivation of latent HIV with single LRAs has produced disappointing *in vivo* outcomes, with little to no measurable impact on plasma viremia.^100^ This challenge may be even more pronounced in the CNS, where viral populations can be more heterogeneous and drug exposure may be constrained. Consequently, leading investigators have argued that effective reservoir depletion will require a combination of approaches, pairing LRAs with passive immune-targeting modalities such as bNAbs.^10^ In that context, we highlight candidate LRAs that could be incorporated into such combination regimens.

### Immune-Based Strategies Targeting CNS Reservoirs: Broadly Neutralizing Antibodies (bNAbs) and Viral Rebound Control

Immune-based approaches, including bNAbs, have gained traction due to their potential to control viral rebound in infected cells. However, CNS delivery remains a critical challenge for these therapeutics. Nevertheless, bNAbs have emerged as a potent immunotherapeutic modality against HIV-1.^101^ These monoclonal antibodies recognize conserved epitopes on the HIV-1 envelope and thus exhibit broad cross-clade activity (Figure 4).^35,102^ Detailed structure–function analyses, including epitope mapping, have identified key regions of vulnerability on the virus, such as the V3-glycan supersite, the V1/V2 apex of the gp120 trimer, and the membrane-proximal external region (MPER) at the base of gp41 (Figure 4).^35,102^

**Figure 4:**
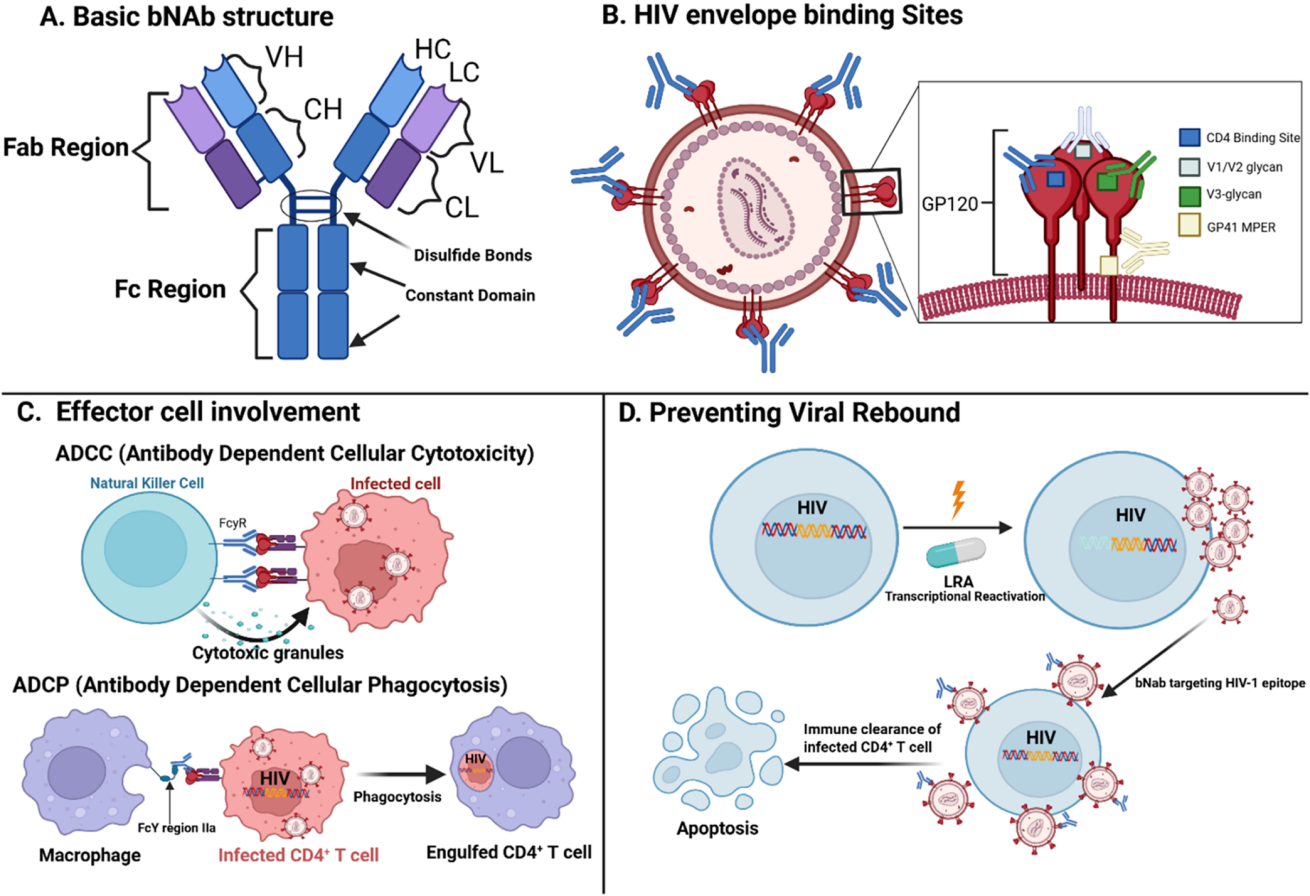
Broadly Neutralizing Antibodies (bNAbs) and Control of HIV Viral Rebound. (A) Schematic representation of a bNAb, highlighting key structural components including the variable region, fragment antigen-binding (Fab) region, and fragment crystallizable (Fc) region. (B) Illustration of the HIV envelope glycoproteins gp120 and gp41, with specific epitopes targeted by individual bNAbs. These epitopes include regions within the variable loops (V2, V3), glycan-dependent sites, and the membrane-proximal external region (MPER). (C) Immune effector cells involved in antibody-mediated clearance mechanisms, specifically antibody-dependent cellular cytotoxicity (ADCC) and antibody-dependent cellular phagocytosis (ADCP), demonstrate how bNAbs facilitate immune recognition and subsequent elimination of HIV-infected cells. (D) Schematic showing bNAb’s binding to HIV-1 virions following transcriptional reactivation of integrated, previously latent provirus in CD4^+^ T-cells. LRA: Latency reversal agent, LPA: Latency promoting agent, bNAb: Broadly neutralizing antibody, ADCC: Antibody-dependent cellular cytotoxicity, ADCP: Antibody-dependent cellular phagocytosis, VH: Variable Heavy, VL: Variable Light, CH: Constant Heavy, CL: Constant Light, Fab: Fragment antigen binding domain, FcyR: Fc-γ receptor, GP120: Glycoprotein 120. Created with BioRender.com.

Two main mechanisms of action have been largely associated with the therapeutic action of bNAbs. (1) Fab-mediated neutralization occurs, in which the fragment antigen-binding (Fab) region of bNAbs can directly neutralize free virions by blocking their attachment or fusion to target cells.^35^ (2) Fc-Dependent Effector Functions: beyond direct neutralization, the fragment crystallizable (Fc) region can engage Fcγ receptors (FcγRs) on macrophages, NK cells, and other leukocytes.^82,102^ This can initiate antibody-dependent cellular phagocytosis (ADCP) or antibody-dependent cellular cytotoxicity (ADCC), promoting the clearance of infected cells expressing HIV envelope on their surface (Figure 4).^82,102^ Moreover, Fc-FcγR interactions can stimulate dendritic cells, amplifying antigen processing and T-cell priming.^82,102^

To underscore this, Wen et al. found that bNAbs are highly effective at suppressing systemic infection with simian-human immunodeficiency virus (SHIV) in infected infant nonhuman primates (NHPs). However, there is currently a lack of studies evaluating whether bNAbs reach the CNS and whether this may be a limiting factor for a cure.^103^ An approach to improve BBB permeability was demonstrated by Wen et al., who designed zwitterionic nanocapsules that enhance the delivery of bNAbs and advance their ability to penetrate the CNS.^103,104^ Within the brain capillary endothelial cells, both choline transporters (ChTs) and nicotinic acetylcholine receptors (nAChRs) are widely expressed.^103^ Thus, the nanocapsules are designed to incorporate both choline and acetylcholine analogs on the surface of a polymer shell, which interacts with choline and nicotinic acetylcholine transporters that mediate transcytosis of the nanocapsules, encouraging penetration into the BBB.^103^ Despite the nanocapsule successfully prolonging the half-life of the PGT121 bNAb and enhancing its CNS delivery in infant NHPs, PGT121 alone was ineffective in eliminating established viral reservoirs.^103^

#### bNAbs and Post-Latency Viral Rebound

A central challenge in “shock and kill” strategies is viral reactivation, which can induce toxicity once latent reservoirs are reactivated. It is crucial to note that bNAbs cannot directly target latently infected cells because they do not express the viral envelope glycoprotein on their surfaces. Therefore, bNAbs rely on the “shock” component of the strategy to clear infected cells. In latently infected cells, bNAbs may mitigate toxic levels of viral rebound by neutralizing newly produced virions and facilitating the destruction of reactivated cells (Figure 4D).^105^ Specifically, preclinical findings have demonstrated that bNAbs can enhance the elimination of HIV-1-infected cells by augmenting innate immune responses.^106^ Combined with LRA’s, bNAb-antigen complexes can form and bind to plasmacytoid dendritic cells via Fc γ receptors.^106^ This leads to the cross-presentation of viral antigens on the major histocompatibility complex (MHC) class I molecule, resulting in HIV-1-specific CD8^+^ T-cell-mediated killing.^106^

Combining the two may boost HIV-1-specific immunity and eliminate infected cells that present viral peptides on their surface.^106^ In one notable study, researchers administered a combination of bNAbs (3BNC117, 10-1074, and PG16) alongside multiple viral inducers, including Vorinostat (an HDAC inhibitor), I-BET151 (a BET protein inhibitor), and an immune checkpoint inhibitor, αCTLA-4, within HIV-infected humanized mice.^105^ Among 23 mice that initially suppressed viremia with antibody therapy, only 10 (43%) exhibited rebound after treatment discontinuation, whereas the remaining 57% remained aviremic, a significantly lower rebound rate than seen with antibody therapy alone or single inducers.^105^

Another study by Julg et al. evaluated the safety and antiviral effects of a triple combination of three bNAbs, conducted in an open-label, two-part study that administered a single dose of the combination. The end points in part 1 of the study include safety, tolerability, and pharmacokinetics. Meanwhile, end points in part 2 included antiviral activity following ART discontinuation, changes in CD4^+^ T-cell counts, and development of HIV-1 sequence mutations associated with bNAb resistance.^107^ They found that the bNAb treatment was generally safe and well-tolerated. In the second arm of the study, 83% of participants maintained virologic suppression for at least 28 weeks.^107^ One of the central challenges associated with bNAb’s is the limited penetration into central nervous system infections.^103^

Although there is much promise for immunotherapy in HIV cure strategies, penetration is typically limited by the BBB, with bNAb levels in the CSF being only 0.1% of the blood concentration after administration.^103^ This creates challenges when antibodies must achieve high concentrations to elicit therapeutic effects. One broader effort to overcome challenges in CNS delivery includes the development of nanotechnology, which has shown promise for delivering macromolecules to the CNS.

#### Epigenetic and Chromatin Remodeling Factors

As discussed in the preceding sections on HIV transcriptional control, the epigenetic landscape at the proviral integration site, including histone acetylation status, DNA methylation, and local chromatin architecture, is a principal determinant of whether HIV-1 remains transcriptionally silent or becomes reactivated.^108^

These mechanisms provide the rationale for a major class of LRAs that directly target the host’s epigenetic machinery, rather than relying on broad immune activation. Many established LRAs, including TLR, PKC, and IL-15 agonists, function by stimulating immune cell activation,^109^ which broadly remodels the epigenetic environment and drives production of transcription factors such as NF-κB and NFAT that engage the HIV LTR.^40^ However, while these immune activators may be highly effective *in vitro*, they have significant risks of inflammatory and autoimmune toxicity *in vivo*.^109^ This limitation has motivated the development of LRAs that target epigenetic-modifying proteins independently of immune activation, which may offer a safer therapeutic window for latency reversal.^54^

Agents that directly target the host epigenetic machinery account for a substantial proportion of LRAs under investigation.^100^ Vorinostat, one of the first LRAs to enter clinical trials, has produced conflicting evidence regarding its efficacy. Some studies reported increased HIV DNA/RNA levels in the periphery, while others observed no significant change. Notably, as described earlier, Vorinostat achieves significant BBB penetration and can inhibit class I and II HDACs, promoting a relaxed chromatin state at the HIV LTR.^110^ However, the inconsistent clinical results highlight a broader challenge in the field: the lack of reliable tools to quantify latency reversal. There is currently no straightforward method to quantify HIV reservoirs in tissues without biopsy, and biopsies are subject to sampling bias that may not capture the full extent of HIV within a given anatomical site. Post-mortem tissue sampling can provide a more comprehensive assessment but has inherent limitations in clinical relevance and interpretability. Even in peripheral blood, measuring latent HIV by qPCR can be technically challenging and may provide imprecise estimates of reservoir size. Emerging assays, such as the intact proviral DNA assay (IPDA), should be incorporated further to assess peripheral latent reservoirs in these studies.^111^

Beyond histone deacetylation, DNA methylation at the LTR represents a complementary mechanism that reinforces proviral silencing. Methylation at two CpG island sites flanking the HIV transcription start site recruits heterochromatin-inducing factors, including Methyl-CpG binding domain protein 2 (MBD2) and HDAC2.^112^ The cytosine methylation inhibitor 5-aza-2′-deoxycytidine (5-azadC) disrupts this repressive complex, shifting the local environment toward euchromatin. Importantly, 5-azadC has demonstrated potent latency-reversal activity when combined with SAHA *in vitro* and *ex vivo*, suggesting that simultaneous targeting of multiple epigenetic silencing mechanisms may be necessary for effective reactivation.^113^ Clinical trials using 5-azadC specifically for HIV latency reversal have not yet been conducted, although the compound is currently under investigation in oncology settings.^76^ A summary of latency reversal agents and their known mechanisms is provided in Table 1.

Finally, BET bromodomain inhibitors offer a mechanistically distinct approach to reversing epigenetic latency. As introduced in the BBB-penetrant LRA discussion above, JQ1 competitively displaces BRD4 from the P-TEFb complex, thereby freeing P-TEFb for Tat-mediated transcriptional elongation.^84,114^ What makes JQ1 particularly noteworthy in the LRA space is its paradoxical ability to suppress T-cell activation and exert anti-inflammatory effects while simultaneously promoting HIV transcription.^114^ This decoupling of immune activation from latency reversal is a highly desirable property, as it may mitigate the inflammatory toxicity associated with other LRA classes. JQ1 has not yet been tested in clinical trials for HIV latency reversal, although structurally related BET inhibitors are currently being evaluated in oncology trials (Figure 5).^114^

**Figure 5:**
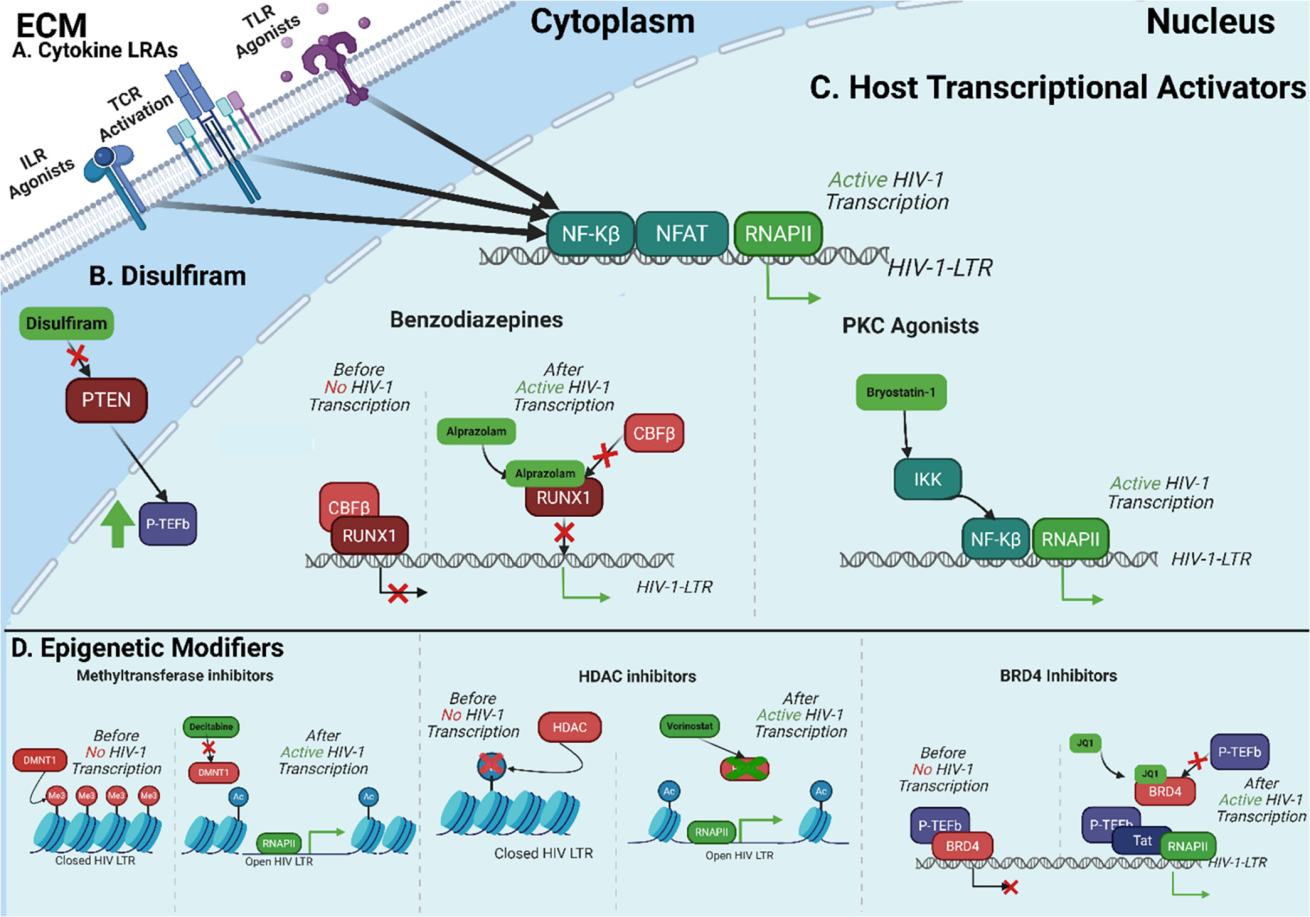
Overview of HIV Latency Reversal Agents. (A) Cytokine reactivation of HIV transcription is generally mediated through increased NF-kB and NFAT binding of the LTR, recruiting RNAPII. (B) Disulfiram can block PTEN/AKT signaling by inhibiting phosphatase, removing phosphate from PIP3. This increases P-TEFb, which mediates Tat-dependent HIV transcription. (C) LRAs can activate host-mediated transcription of HIV. PKC agonists increase NF-kB activity by enhancing its binding to the LTR. Benzodiazepines disrupt CBFβ binding to RUNX1, increasing transcription. (D) BRD4 inhibitor competitively block the binding of P-TEFb to BRD4, allowing P-TEFb to bind with Tat and complete Tat-dependent transcription. Methyltransferase inhibitors block the addition of methyl groups, leading to a more transcriptionally active epigenetic environment. HDAC inhibitors block histone deacetylation, leading to a more transcriptionally active epigenetic environment. ECM: Extracellular matrix, DMNT1: DNA methyltransferase 1, HDAC: Histone Deacetylase, RNAPII: RNA Polymerase II, NFAT: Nuclear Factor of Activated T-cells, NF-kB: Nuclear factor-kappa B, IKK: I kappa B kinase, P-TEFb: Positive Transcription Elongation Factor b, BRD4: Bromodomain-containing protein 4. Created with BioRender.com.

#### The Wnt/β-Catenin Pathway in HIV Latency

Another interesting approach to reversing HIV latency involves modulating the Wnt/β-catenin pathway. Although inhibitors have failed to decrease reservoir size in isolation, this pathway may play an essential role in future LRA combinations. The role of the Wnt/β-catenin pathway in glial cells and neurons is complex; however, it is known to be a key regulator of stem cell proliferation and differentiation. Beyond these traditional roles, there is increasing evidence that the canonical Wnt/β-catenin pathway is repressed during HIV latency.^115–120^

Early investigations into this finding proposed that HIV-1 Clade B Tat, but not Clade C Tat, can physically bind and sequester T-cell factor 4 (TCF-4), a transcription factor that partners with β-catenin. This binding was shown to block the transcription of Wnt target genes and HIV. When the cysteine-rich domain or core domain of Tat was mutated, binding to TCF-4 was abolished, supporting this theory.^115^

These findings were most significant in astrocytes, due to the naturally higher Wnt pathway activity in these cells compared to T-cells. Beyond inducing HIV latency, the canonical Wnt pathway is key for neuronal health, and dysregulation by HIV could contribute to HIV-associated neurocognitive disorders.^116^

Further investigation confirmed the exact mechanism: Tat binds TCF-4 to repress HIV transcription in CD4^+^ T-cells. In a study by Mavigner et al., the β-catenin inhibitor PRI-724 was evaluated in SIV-infected rhesus macaques. Unlike astrocyte studies, this work aimed to address a different aspect of HIV latency: by forcing infected stem cell-like memory T-cells to differentiate, the cells would become shorter-lived, and the reservoir would eventually dwindle. PRI-724 blocks the interaction between β-catenin and the CREB-binding protein (CBP), thereby favoring β-catenin binding to p300 and promoting T-cell differentiation. This therapy significantly reduced Stem Cell Memory and Central Memory CD4^+^ T-cells; however, the total reservoir size did not decrease.^119^

This suggests that while β-catenin modulation is a promising approach, it may be most effective as a component of combination therapies. By preventing the proliferation of infected cells or forcing their differentiation, such therapies could prevent reservoir replenishment while other LRAs allow infected cells to be detected by the immune system and cleared.

#### Highlighting RUNX1, an Emerging Target

One particularly compelling regulator of HIV transcription is RUNX1, a transcription factor implicated in hematopoiesis and HIV latency. Inhibition of RUNX1 may offer novel paths for reactivating silent proviruses. Among PLWH, psychological stress often arises from living with a stigmatized illness.^121^ Benzodiazepines are frequently prescribed to manage anxiety and insomnia. Longitudinal studies have reported an association between benzodiazepine use and worsened HIV-associated neurocognitive disorders, prompting research into the role of benzodiazepines in HIV transcription.^122^ For example, alprazolam, commonly known as Xanax, has been shown to enhance HIV transcription by inhibiting RUNX1 and activating Signal Transducer and Activator of Transcription 5 (*STAT5*), thereby driving T cell activation independently of cytokines such as IL-2.^72^

Both illicit drugs and misused therapeutics exacerbate CNS disease by increasing neuroinflammation and neuropathology. Among these are benzodiazepines (BDZ) such as alprazolam (Xanax) and diazepam (Valium). More than 30 million Americans use these therapeutics to treat anxiety and panic disorders, insomnia, alcohol withdrawal, and other symptoms.^123^ While generally thought of as therapeutics, BDZ misuse accounts for 15–20% of overall use, most often due to nonprescription use or use more than the clinical dosage for recreational purposes.^74,123^

The use of BDZ is particularly high in PLWH, as these drugs are often used to ameliorate comorbidities in this population. Despite their high misuse liability^74^ and the growing use of these drugs by PLWH, BDZ is heavily understudied compared with stimulants and opiates. This is important because BDZ can exacerbate neuropathology and neurocognitive dysfunction through disruptions in reward circuitry and contributions to a neurotoxic environment.^73^ A study showed that PLWH who use BDZ, specifically alprazolam and diazepam, had a significantly increased risk of developing neurocognitive impairment, particularly in global function, processing speed, and motor domains.^122^

The mechanisms by which BDZs exacerbate HAND are poorly understood; however, our data suggest that BDZs mediate changes in the chromatin environment that increase transcriptional activity by inhibiting a transcription factor called RUNX1.^124^ This protein forms a heterodimer with its binding partner, Core-Binding Factor β (CBFβ), to bind DNA efficiently and to regulate transcription by recruiting additional transcription factors. We and others have demonstrated that RUNX1 acts on the HIV LTR to regulate T-cell transcription, and that RUNX1 activity may be modulated by other viral proteins, such as the HIV viral infectivity factor (Vif).^125^ RUNX1 is also present in microglia and required for many microglial functions.^126^

We have shown that RUNX1 levels in patients correlate with HIV viral load,^127^ that RUNX1 inhibition by alprazolam induces *STAT5* activation,^72^ and that even low concentrations of clinically prescribed BDZs, including alprazolam, inhibit RUNX1 function and reactivate HIV in latently infected cells in tissue culture.^128^ The overlap between BDZ use and HIV infection highlights the potential risks and therapeutic upsides of BDZ in the context of HIV, showing a clear need to determine if strategies to silence HIV in the CNS are durable in the face of BDZ exposure. RUNX1 may affect HIV transcription in multiple ways, including binding the NF-κB p50 subunit^121^ and potentially influencing TLR4-driven inflammation and HIV gene regulation. NF-kB p50 is central to latency, promoting HDAC recruitment and transcriptional repression when p50 forms a homodimer, whereas p50/p65 heterodimers activate HIV transcription. Overexpression of RUNX1 and its binding partner, CBFβ, in cell models significantly dampened HIV expression.^125^

A nonpsychiatric benzodiazepine, Ro5–3335, used in conjunction with suberoylanilide hydroxamic acid (SAHA), synergistically enhanced HIV transcription with minimal impact on T-cell activation.^124^ An exploration of RUNX1’s impact on Tat-dependent transcription, using a TR-FRET assay, demonstrated that Tat could competitively inhibit the RUNX1-CBFβ interaction.^124^ RUNX1 can bind to Tat with high affinity and inhibit Tat-mediated transcription, signifying RUNX1’s primary mechanisms as inhibition of Tat transactivation.^125^ In addition to influencing T-cell transcriptional machinery, RUNX1 also operates in myeloid cells, which modulate gene expression and can further shape HIV pathogenesis.^129^

#### Stem Cell Transplants and Gene Editing Strategies

To date, a total of seven individuals have been cured or are in long-term remission of HIV infection. In most of these cases, the patients received allogeneic hematopoietic stem cell transplants from donors carrying a rare genetic mutation called CCR5-delta 32, which prevents HIV from entering cells. Some of these patients living remission-free, such as the Geneva patient, did not have the CCR5-delta 32 mutation in their stem cells, questioning the importance of this mutation relative to the new donor’s immune system detecting and clearing old HIV-infected cells.

Although promising, this approach remains incredibly risky with a mortality rate of around 10% due to Graft-versus-Host Disease, making it undesirable for PLWH, as current ART is highly effective. Research has been moving toward autologous gene-editing approaches, in which a patient’s own stem cells are harvested, genetically edited with Zinc Finger Nucleases or CRISPR-Cas9 to mutate the CCR5 gene, and reinfused into the patient. The early trials with ZFNs and CRISPR proved safe; however, the number of cells edited did not make a significant difference, and viral rebound occurred soon after.^130,131^ In 2021, a group from the University of Pennsylvania investigated electroporation to deliver the gene-editing payload. This showed greater mRNA delivery efficacy and a higher percentage of edited T-cells; however, the median CCR5 editing rate with ZFNs remained at only 24%.^132^

Newer technologies such as EBT-101 from Excision BioTherapeutics aim to directly excise the integrated HIV provirus from the host genome by delivering CRISPR-Cas9 *in vivo* via adeno-associated viral vectors. As of 2024, the five participants tolerated EBT-101 safely; however, the three patients who discontinued ART experienced viral rebound and had to restart ART.^133^

Future experiments should either confirm that the Graft-versus-Viral effect was responsible for the seven patients’ cure, or seek more effective ways to mutate CCR5 *ex vivo*. These trials continue to teach us the intricacies of HIV and will continue to guide us toward a cure.

## Toll-like Receptors in HIV-1 Latency Reversal

Toll-like receptors (TLRs) are pattern recognition receptors (PRRs) that play a pivotal role in detecting pathogen-associated molecular patterns (PAMPs) and damage-associated molecular patterns (DAMPs).^134^ TLRs are expressed in several innate and adaptive immune system cells, including macrophages, granulocytes, T-cells, B cells, Natural Killer (NK) cells, and mast cells.^134^ This includes antigen-presenting cells (APCs) such as dendritic cells. TLRs 1, 2, 4, 5, 6, and 10 are localized primarily to the cell surface, whereas TLRs 3, 7, 8, and 9 reside in endosomal compartments (Figure 6).^134^ Cell surface TLRs recognize various bacterial components, including flagellin, lipopolysaccharides, peptidoglycans (PGN), and lipoproteins (Figure 6).^135^ When TLRs sense microbial motifs, they trigger intracellular signaling cascades that culminate in the expression of pro-inflammatory cytokines and transcription factors, notably NF-kB, which can also reactivate latent HIV (Figure 6).^134^

**Figure 6:**
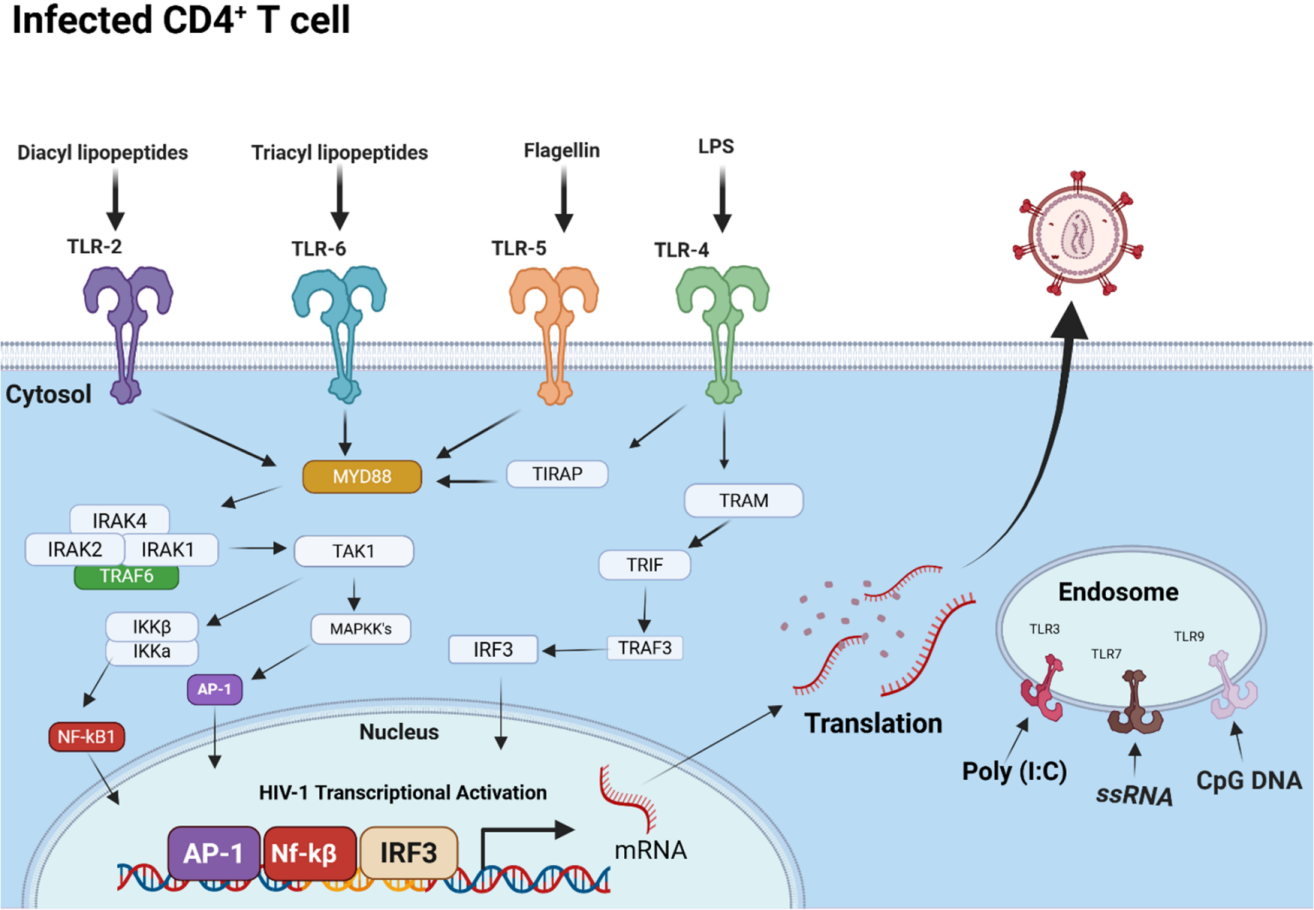
TLR-mediated HIV Latency Reversal. A schematic illustration of the Toll-like receptors (TLRs) involved in HIV latency reversal in latently infected CD4^+^ T-cells. TLRs 2, 4, 5, and 6 are depicted on the cellular surface, whereas TLRs 3, 7, and 9 are localized within endosomal compartments. Pathogen-associated molecular patterns (PAMPs), including microbial and viral motifs, interact with these TLRs, initiating intracellular signaling cascades. Activation of these pathways leads to nuclear translocation of transcription factors such as AP-1, NF-κB, and IRF3, facilitating their binding to the HIV 5′ LTR region and resulting in transcriptional reactivation of latent HIV. TLR: Toll-Like Receptor, NF-kB: NF-Kappa β, LPS: Lipopolysaccharide. Created with BioRender.com.

Glial cells (microglia and astrocytes) express toll-like receptors (TLRs) that recognize various PAMPs, including TLR2 and TLR3 agonists such as peptidoglycan and double-stranded RNA.^136^ This initiates the release of pro-inflammatory mediators, including proinflammatory cytokines (TNF-α, IL-1β), chemokines (MIP-2), and reactive oxygen/nitrogen species (O_2_^–^, NO).^136^ Subsequently, this contributes to the influx of peripheral immune cells into the CNS (e.g., neutrophils, lymphocytes, and macrophages) by increasing BBB permeability.^136^ This neuroinflammatory response can help eliminate invading pathogens by recruiting and mobilizing leukocytes to affected tissues.^136^

### Evidence Linking TLRs to HIV Reactivation

Early clinical observations reported increases in plasma viral load in PLWH during opportunistic bacterial infections.^134^ It was hypothesized that PAMPs indirectly transactivate the HIV LTR promoter, thereby promoting viral transcription.^134^ Consistent with this model, purified protein derivative (PPD) from *Mycobacterium tuberculosis* induced viral mRNA expression in HIV-infected monocytes.^134^

Further studies confirmed that mycobacterial components, such as mannosylated LAM (ManLAM), activated HIV in Jurkat T cells, primarily via protein kinase pathways leading to NF-κB nuclear translocation.^134^ Similarly, flagellin, a structural component of bacterial flagella and a TLR5 agonist, reactivated HIV in J-Lat cells.^134^ This is mainly because this agonist induced NF-kB.^137^ Moreover, in one experiment, Thibault et al. transiently transfected Jurkat cells with pNF-κB-LUC, pκB-TATA-LUC, or pLTRX-LUC.^137^ These plasmid constructs encode a luciferase reporter.^137^ The authors showed that flagellin stimulation increased luciferase activity, consistent with NF-kB activation.^137^

These findings highlight two complementary modes of TLR-mediated latency reversal. The first is an indirect mechanism, in which TLR agonists activate bystander immune cells (e.g., dendritic cells, macrophages), leading to the secretion of cytokines and other soluble mediators that subsequently induce HIV transcription in latently infected CD4^+^ T-cells.^134^ The second is a direct mechanism in which specific TLR agonists act on latently infected CD4^+^ T-cells themselves, triggering intracellular signaling cascades that often converge on NF-kB and promote viral transcription.

Moreover, TLR7 agonists, including GS-9620, are among the most studied compounds in this class. It induces HIV RNA release in cells from HIV-infected individuals on ART by activating plasmacytoid dendritic cells (pDCs), which then release type I interferons and other cytokines.^134,138^ In one study, Alvarez-Carbonell et al. investigated the effect of TLR ligands on the reactivation of proviral HIV in human models of latently infected microglial cells. Specifically, the goal was to evaluate several agonists, including LPS (a TLR4 agonist), flagellin (a TLR5 agonist), and FSL-1 (a TLR6 agonist), all of which reactivated HIV to a lesser extent. Pam3CSK4 (and TLR2/1 agonist) and HKLM (a TLR2 agonist) weakly reversed HIV latency. These TLR agonists triggered a direct mechanism that induced NF-kB.

In contrast, the TLR3 agonist poly­(I:C) induced the virus through a mechanism mediated by the IRF3 transcription factor. To a lesser extent, TLR4, 5, and 6 agonists reactivated HIV significantly in these latently infected microglial cells through NF-kB nuclear translocation. The engagement of TLR3 with agonist poly­(I:C) caused the strongest HIV reactivation in human microglial cells.^139^

It is important to note that ten different TLRs have been identified as endogenous in humans, as listed in Table 2. TLR2 agonists reactivate HIV by directly inducing NF-kB in memory CD4^+^ T-cells.^138^ Meanwhile, TLR5, TLR8, and TLR9 agonists have all been shown to increase HIV gene expression within *in vitro* models.^138^ Because TLR agonists can boost immune effector functions (e.g., the release of interferons) and reactivate latent viruses, they represent a promising adjunct to current latency-reversal approaches. TLR7 agonists, such as GS-9620, can complement other LRAs or immunotherapies by enhancing innate and adaptive immune responses against newly infected HIV cells.^134,138^ Future research, including *in vivo* studies and clinical trials, will be critical to fully understand the efficacy, dosing, and safety of TLR-based strategies in both peripheral and CNS HIV reservoirs.

**Table 2:** CNS Toll-Like Receptor Agonists

**receptor**	**agonist**	**refs**
**TLR 1**	lipoproteins	^135,136,140^
	PAM3CSK4	
**TLR 2**	SMU-Z1	^135,141^
	Aβ	
	biglycan	
	endoplasmin (HSP90B1)	
	HeatShockProteins (HSP60, HSP70)	
	HMGB1	
	hyaluronan	
	monosodium urate crystals	
	α-synuclein	
	surfactant protein A	
	fibronectin	
	versican	
**TLR 3**	polyinosinic: polycytidylic acid Poly (I:C).	^135,139^
	bacterial rRNA.	
**TLR4**	lipopolysaccharide (LPS).	^135,139^
	Aβ	
	αA-crystallin, αB-crystallin	
	endoplasmin (Hsp90b1)	
	fibronectin	
	heparan sulfate	
	HSP60	
	HSP70	
	HSP72	
	hyaluronan	
	lysozyme	
	monosodium urate crystals	
	peroxiredoxin 1	
	resistin	
	S100 protein	
	surfactant protein A	
	tenascin C.	
**TLR5**	flagellin.	^139^
**TLR6**	fibroblast-stimulating lipopeptide (FSL-1).	^139^
**TLR7**	imiquimod	^135,138,139^
	gardiquim	
	resiquimod	
	GS-962	
	miRNA: (Let-7B, miR-146a-5p, miR-340–3p, miR-132–5p).	
**TLR8**	imiquimod	^135,139^
	gardiquimod	
	resiquimod	
	ssRNA40	
	miRNA: (miR-27, miR-21, miR-340–3p and miR-132–5p).	
**TLR9**	ODN2006.	^135,139^
	DNA.	
	mtDNA.	
	chromatin-IgG complex.	

### Implications for HIV Cure Strategies

Because TLR agonists can boost immune effector functions (e.g., interferon release) and reactivate latent viruses, they represent a promising adjunct to current latency-reversal approaches. TLR7 agonists, such as GS-9620, could complement other LRAs or immunotherapies by enhancing innate and adaptive immune responses against newly revealed HIV-infected cells.^136,142^ Future research, including *in vivo* studies and clinical trials, will be critical to fully understand the efficacy, dosing, and safety of TLR-based strategies in both peripheral and CNS HIV reservoirs.

### Pharmacokinetics and BBB Penetration

HIV reservoirs persist in various compartments, including peripheral blood, lymph nodes, bone marrow, the gastrointestinal tract, and, most critically, the central nervous system (CNS). Because the BBB restricts the penetration of many therapeutics, developing LRAs that can effectively cross this barrier is crucial for targeting CNS reservoirs.

Currently, few LRAs have been validated for CNS efficacy. Among them, benzodiazepine RUNX1 inhibitors, such as Ro5-3335, are notable for their lipid-soluble, bicyclic heterocyclic structure, which facilitates BBB crossing. Nevertheless, evidence remains sparse regarding their long-term pharmacokinetics, optimal dosing, and *in vivo* efficacy in reactivating latent HIV, specifically within CNS cell types (e.g., astrocytes and microglia).

### Safety, Neurotoxicity, and Off-Target Effects

Reactivating HIV within the CNS poses a double-edged sword. Contrarily, latent viruses must be exposed to achieve immune clearance. In contrast, uncontrolled viral replication in the brain could trigger neuroinflammation and neuronal damage. To effectively manage viruses in these secluded reservoirs, “killing” or clearing infected cells postreactivation must be optimized before clinical use.^14^

1. **Neurotoxicity and BBB Considerations:** Although BBB penetration for LRAs is desirable, off-target effects in the CNS can compromise neuronal health. Because neurons exhibit limited regenerative capacity, even temporary neurotoxicity can lead to long-term functional deficits. Thus, safer, more specific molecules are needed.
2. **Known Toxicity Profiles:** Multiple LRAs have demonstrated toxicity across various cell types, including benzodiazepines, Bryostatin-1, JQ1, and disulfiram. These compounds exhibit duality: while they might protect against HIV-associated neurocognitive impairment, they can also induce small molecule-mediated neurotoxicity if not carefully dosed.^85^

### Specific Examples

#### JQ1

Linked to cell cycle arrest and differentiation defects in human umbilical cord mesenchymal cells,^85^ JQ1 increases Caspase 9 and Cytochrome C, suggesting potential neurotoxicity in neuronal derivatives.

#### Vorinostat

Although generally considered safe at dosages of 400 mg, widespread gene expression changes may be responsible for side effects, including fatigue and weakness, hair loss, and skin irritation.^143^

#### Benzodiazepines

Long-term use correlates with an increased risk of dementia. Common side effects are sedation, ataxia, poor concentration, and irritability, highlighting the need for novel RUNX1 inhibitors with reduced neurotoxic effects.^74^

#### Disulfiram

There are countering claims for the toxicity of disulfiram, and at doses of 1–3 g, disulfiram demonstrates considerable harm to neuronal and liver cells, with some deaths occurring from hepatotoxicity.^94^ However, a 250 mg/day dosage provided little evidence of toxicity.^94^ A recent clinical trial found that combining Vorinostat (400 mg) and disulfiram (2 g) significantly increased plasma HIV RNA levels. However, the trial was suspended due to substantial neurotoxicity.^69^ Therefore, dosage and efficacy must be carefully considered when deciding on LRA dosages.

## Emerging Approaches and Future Directions

LRAs have been actively pursued as a cure strategy for over a decade. However, significant challenges are associated with current LRAs. For example, the endogenous cytotoxic T lymphocyte (CTL) response needed to target infected cells for clearance is often ineffective.^144^ Various HDAC inhibitors, including romidepsin, panobinostat, and SAHA, were evaluated for their suppressive effects on IFN-γ production in HIV-specific antigen-stimulated CD8^+^ T-cells.^145^ The HDAC inhibitors were utilized to treat CD8^+^ T-cell clones that were isolated from two ARV-treated HIV-infected subjects.^145^ All compounds were administered at pharmacologically relevant concentrations. For two compounds (panobinostat and SAHA), inhibition of cytokine production was dosage-dependent, whereas romidepsin was not.^145^ All tested HDAC inhibitors suppressed HIV-specific IFN-γ production from *ex vivo* CD8^+^ T-cell samples.^145^

Additionally, some concerns exist that LRAs have some neurotoxic effects.^66^ It has been observed that various LRAs are not exclusively reactivating HIV-infected cells, leading to immune activation and bystander cell toxicity.^54,62,95^ In addition to toxicity, LRA’s effectively induce viremia; however, this strategy still cannot eliminate viral reservoirs in clinical trials.^146^ Thus, there are increasing investigations into the use of novel compounds associated with immune regulation, such as bNAbs and cellular therapies, including Chimeric Antigen Receptor (CAR) T-cells.^146^ These therapies have made significant progress in the cure of HIV, although certain limitations, such as BBB penetration, should be addressed.^147^

In contrast to bNAbs, CAR-T cells have been shown to cross the BBB;^136^ however, whether they have detrimental off-target effects in the CNS is unknown. Neurotoxicity is a common side effect associated with the use of CAR-T cells for cancer treatment.^148^ New generations of anti-HIV CAR-T cells have been developed, including multispecific “duo CAR-T” cells with two CAR molecules that bind to the HIV-1 glycoprotein, comprising multiple HIV binders expressed on the T-cell surface.^149^ These T-cells are transduced with a single lentiviral vector (LV) and engineered to target multiple sites on the HIV-1 viral envelope glycoprotein, thereby increasing their breadth and potency compared to mono-CAR T cells.^149^ Data using duo CAR-T have demonstrated potent reductions of up to 99% of HIV-1-infected cells *in vitro*. *In vivo* studies using HIV-1-infected mouse models have also shown the elimination of up to 97% of HIV-infected cells.^149^ It is essential to note that cellular therapies aimed at enhancing immunity may potentially lead to substantial adverse neurological effects.^147^ There are associations with specific neurotoxic effects or neurological adverse events.^149^ Immunomodulatory therapies or engineered T-cells can induce cytokine release syndrome (CRS) and immune effector cell-associated neurotoxicity syndrome (ICANS), both of which are linked to heightened immune effector responses.^148^ However, it has also been reported that ICANS is more closely associated with systemic cytokine release than with target antigen expression in the CNS.^147,148^

Additional research and investigation are required to examine further LRAs in combination with bNAbs or CAR-T cells, particularly regarding their efficacy against HIV-1 harbored in quiescent CD4^+^ T-cells within the CNS.^106^ Moreover, there are virtually no studies evaluating the effects of combining RUNX1 inhibition with existing LRAs, bNAbs, engineered CAR-T cells, toll-like receptor agonists, or other immunomodulatory cell therapies or latency-promoting compounds/strategies. Examining the synergistic effects of these agents may represent an exciting avenue for further exploration. Such combination therapies illustrate the growing consensus that no single approach will suffice. Instead, an array of synergistic interventions is needed to fully reactivate and eliminate HIV reservoirs.

Beyond challenges in therapeutic delivery, the heterogeneity of HIV reservoirs represents a major barrier to cure efforts. This diversity raises fundamental questions, including how to develop interventions that can effectively target distinct reservoir cell types and states across tissue compartments. Collectively, these considerations underscore the need for novel approaches capable of addressing a broad spectrum of viral phenotypes and enabling controlled activation or durable silencing within alternative cellular reservoirs.
